
JOURNAL INFORMATION
==============================
NLM Title Abbreviation: ScientificWorldJournal
Journal ID: ScientificWorldJournal
NLM Journal ID: 101131163
Journal ID: TSWJ

The Scientific World Journal

ISSN: 2356-6140
EISSN: 1537-744X
Publisher: Wiley

ARTICLE INFORMATION
==============================
PMCID: PMC6009688
PMID: 29973845
DOI: 10.1100/tsw.2002.70
Article ID: 397139
Article version: 1
Subjects: Short Report

Some Aspects of Dna-Etbr Interaction Peculiarities

Vardevanyan Poghos O. 1 *
Antonyan Ara P. 1
Minasbekyan Lia A. 1
Karapetyan Armen T. 2
Biophysics Department of Yerevan State University, 375025 Yerevan, Armenia
Physics Department, Yerevan Institute of Architecture and Construction, 375009 Yerevan, Armenia
*Poghos O. Vardevanyan: simvar@arminco.com
Publication date: 2002
Electronic publication date: 2002 Mar 27
Volume: 2
First page: 144
Last page: 145
Copyright: Copyright © 2002 Poghos O. Vardevanyan et al.
Copyright year: 2002
License: This is an open access article distributed under the Creative Commons Attribution License, which permits unrestricted use, distribution, and reproduction in any medium, provided the original work is properly cited.
License URL: https://creativecommons.org/licenses/by/3.0/

==============================
Advances in Gene Technology: The Genome and Beyond – Structural Biology for Medicine (Proceedings of the 2002 Miami *Nature Biotechnology* Winter Symposium)
*TheScientificWorld* 2002, 2(S2), 144–145
ISSN 1532-2246; DOI 10.1100/tsw.2002.70

### SOME ASPECTS OF DNA-ETBR INTERACTION PECULIARITIES

Poghos O. Vardevanyan[1*], Ara P. Antonyan[1], Lia A. Minasbekyan[1], and Armen T. Karapetyan[2]
[1]Biophysics Department of Yerevan State University, 375025 Yerevan, Armenia; [2]Physics Department, Yerevan Institute of Architecture and Construction, 375009 Yerevan, Armenia
E-mail: *simvar@arminco.com

Comparision of theoretical and experimental data of ethidium bromide (EtBr) binding to DNA suggested that this ligand can complex with DNA by several different types of interactions[1,2]. The dependence of temperature transition width from DNA melting curves against the ratio of EtBr/DNA - $r_b$ showed inverted bell-shaped profiles ($0,25 < r_b < 0,55$).

On increasing the EtBr/DNA ratio, the temperature transition width in DNA melting curves decreased, passed through a minimum, and then slightly increased, indicating that EtBr binds to both single- and double-stranded DNA (Fig. 1). In addition, the melting curves showed a dependence on ionic strength, indicating that electrostatic interactions influence the different binding modes. Scatchard analysis of fluorescence binding data of EtBr with DNA detected only intercalation of EtBr into DNA (Fig. 2). However, Scatchard analysis of the absorbance binding data detected the intercalative interaction as well as additional binding modes (Fig. 2). Subtraction of the fluorescence titration data from the absorbance data showed that the additional modes of binding involve nonintercalative interactions between EtBr and both single- and double-stranded DNA (Fig. 2). These results are consistent with previous theoretical and experimental results.

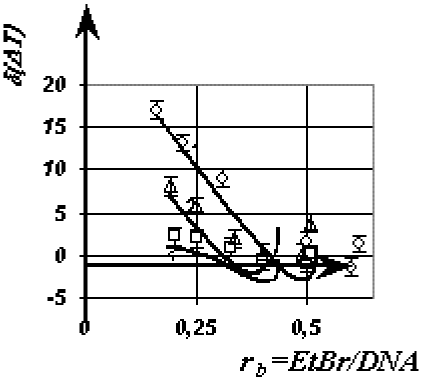

**FIGURE 1.** Melting curves of complexes of DNA-EtBr at ionic strength $\mu_{Na+} = 2,0 \cdot 10^{-3}$ M (curve 1), $\mu_{Na+} = 1,0 \cdot 10^{-2}$ M (curve 2), $\mu_{Na+} = 2,0 \cdot 10^{-2}$ M (curve 3), pH = 7,0. The average square deviation errors are shown.

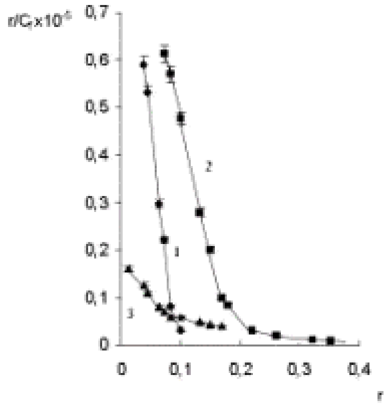

**FIGURE 2.** The binding curves of EtBr with ds-DNA obtained by Scatchard method from fluorescence (1) and absorbtion (2) spectrum at $\mu_{Na+} = 2{\cdot}10^{-2}$ M. The curve (3) is obtained by subtracting curve (1) from curve (2) and correspond to semi-intercalative complex of EtBr with ds-DNA.

### REFERENCES

1. Karapetian, A.T., Mehraiam, N.M., Terzikian, G.A., Vardevanian, P.O., Antonian, A.P., Borisova, O.F., and Frank-Kamenetskii, M.D. (1996) J. Biomol. Struct. Dynam. 14, 275–283.
2. Vardevanyan, P.O., Antonyan, A.P., Manukyan, G.A., Karapetian, A.T., Shchelkina, A.K., and Borisova, O.F. (2000) Mol. Biol. (Rus) 34(2), 310–315.
